# One Health: A new definition for a sustainable and healthy future

**DOI:** 10.1371/journal.ppat.1010537

**Published:** 2022-06-23

**Authors:** One Health High-Level Expert Panel (OHHLEP), Wiku B. Adisasmito, Salama Almuhairi, Casey Barton Behravesh, Pépé Bilivogui, Salome A. Bukachi, Natalia Casas, Natalia Cediel Becerra, Dominique F. Charron, Abhishek Chaudhary, Janice R. Ciacci Zanella, Andrew A. Cunningham, Osman Dar, Nitish Debnath, Baptiste Dungu, Elmoubasher Farag, George F. Gao, David T. S. Hayman, Margaret Khaitsa, Marion P. G. Koopmans, Catherine Machalaba, John S. Mackenzie, Wanda Markotter, Thomas C. Mettenleiter, Serge Morand, Vyacheslav Smolenskiy, Lei Zhou

**Affiliations:** 1 Universitas Indonesia, Depok, West Java, Indonesia; 2 National Emergency Crisis and Disasters Management Authority, Abu Dhabi, United Arab Emirates; 3 Centers for Disease Control and Prevention, Atlanta, Georgia, United States of America; 4 World Health Organization, Guinea Country Office, Conakry, Guinea; 5 Institute of Anthropology, Gender and African Studies, University of Nairobi, Nairobi, Kenya; 6 National Ministry of Health, Autonomous City of Buenos Aires, Argentina; 7 School of Agricultural Sciences, Universidad de La Salle, Bogotá, Colombia; 8 International Development Research Centre, Ottawa, Canada; 9 Indian Institute of Technology (IIT), Kanpur, India; 10 Brazilian Agricultural Research Corporation (Embrapa), Embrapa Swine and Poultry, Concórdia, Santa Catarina, Brazil; 11 Institute of Zoology, Zoological Society of London, London, United Kingdom; 12 Global Operations Division, United Kingdom Health Security Agency, London, United Kingdom; 13 Global Health Programme, Chatham House, Royal Institute of International Affairs, London, United Kingdom; 14 Fleming Fund Country Grant to Bangladesh, DAI Global, Dhaka, Bangladesh; 15 Afrivet B M, Pretoria, South Africa; 16 Faculty of Veterinary Science, University of Kinshasa, Kinshasa, Democratic Republic Congo; 17 Ministry of Public Health, Health Protection & Communicable Diseases Division, Doha, Qatar; 18 Chinese Center for Disease Control and Prevention, Beijing, People’s Republic of China; 19 Molecular Epidemiology and Public Health Laboratory, Massey University, Palmerston North, New Zealand; 20 Mississippi State University, Starkville, Mississippi, United States of America; 21 Erasmus MC, Department of Viroscience, Rotterdam, the Netherlands; 22 EcoHealth Alliance, New York, New York, United States of America; 23 Faculty of Health Sciences, Curtin University, Perth; 24 School of Chemistry and Molecular Biosciences, The University of Queensland, Brisbane, Australia; 25 Centre for Viral Zoonoses, Department of Medical Virology, Faculty of Health Sciences, University of Pretoria, Pretoria, South Africa; 26 Friedrich-Loeffler-Institut, Federal Research Institute for Animal Health, Greifswald-Insel Riems, Germany; 27 MIVEGEC, CNRS-IRD-Montpellier Université, Montpellier, France; Faculty of Veterinary Technology, Kasetsart University, Bangkok, Thailand; 28 Federal Service for Surveillance on Consumer Rights Protection and Human Well-being (Rospotrebnadzor), Moscow, Russian Federation; Boston Children’s Hospital, UNITED STATES

The Severe Acute Respiratory Syndrome Coronavirus 2 (SARS-CoV-2) pandemic once more demonstrated the close connection between humans, animals, and the shared environment. Although still under investigation, the closest relatives of this virus exist in animals, and the factors leading to spillover remain to be fully understood. This interconnectedness again highlighted the need for a One Health approach. Although the One Health concept is not new and has been at the forefront of interdisciplinary and multisectoral discussions for years, there is now an increased interest for this approach to be applied and translated into action. Following a proposal made by the French and German Ministers for Foreign Affairs at the November 2020 Paris Peace Forum, 4 global partners, the Food and Agriculture Organization (FAO), the World Organization for Animal Health (OIE), the United Nations Environment Programme (UNEP), and the World Health Organization (WHO), in May 2021 established the interdisciplinary One Health High-Level Expert Panel (OHHLEP) (https://www.who.int/groups/one-health-high-level-expert-panel) to enhance their cross-sectoral collaboration. The creation of OHHLEP represents a recognition at the highest level of the urgency and complexities surrounding One Health and the intent to take this concept forward into policies and concrete actions.

**Fig 1:**
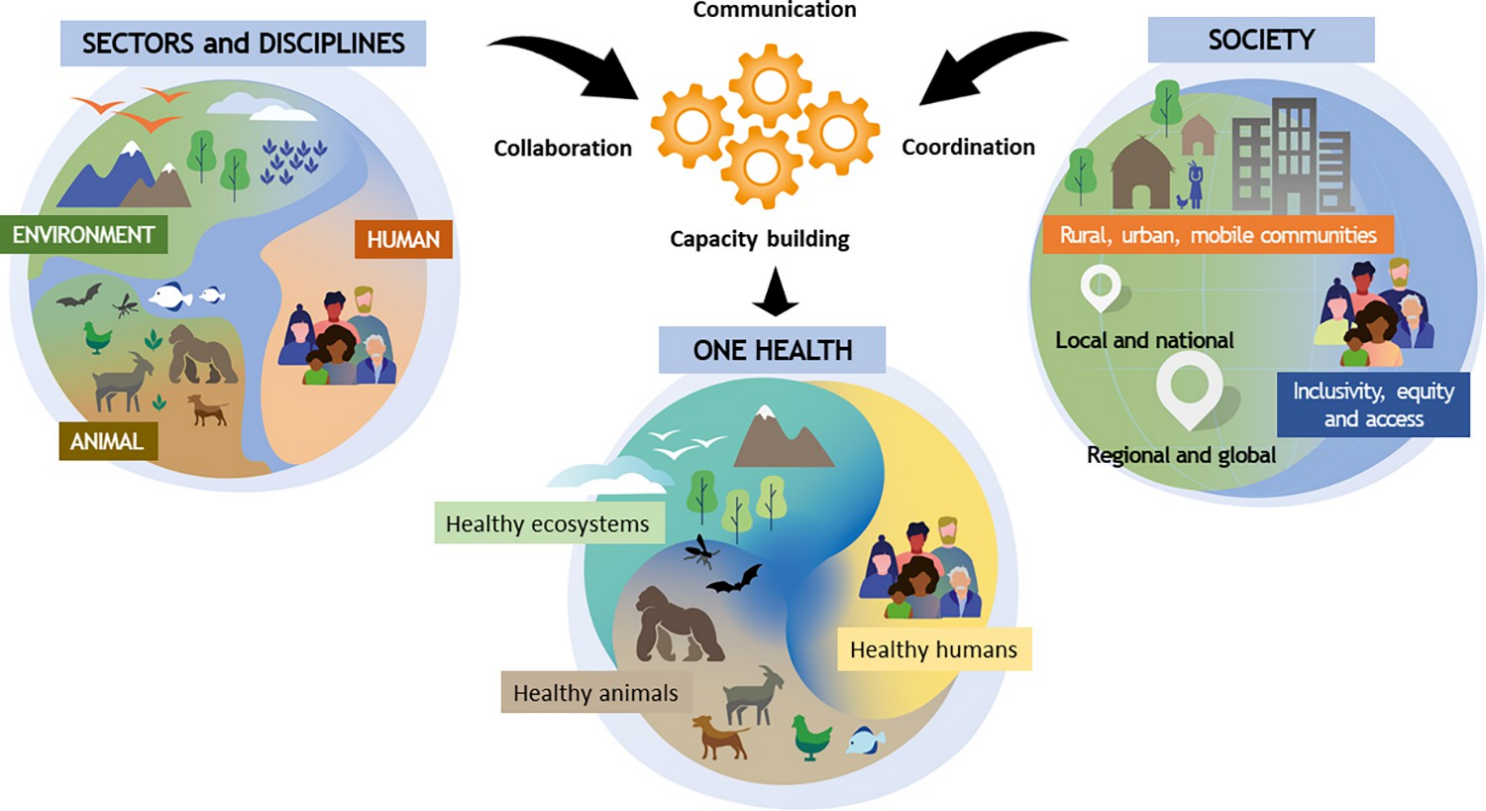
One Health toward a sustainable healthy future as developed by the OHHLEP. OHHLEP, One Health High-Level Expert Panel.

The concept of One Health has been associated with different interpretations in scope and practice. There is no shortage of “One Health” definitions in the published literature and among institutions and organizations. Therefore, an immediate priority for OHHLEP was to develop consensus around a working definition as a solid basis to support a common understanding among the panel members and the partner organizations. It is also relevant to a much broader global audience. Central to this definition is actual implementation, visualized in Fig 1, taking One Health from theory to practice, as highlighted by the 4 Cs: Communication, Coordination, Collaboration, and Capacity building. In applying the One Health view, we also highlight that it is based on several fundamental principles (Box 1), including equity, inclusivity, equal access, parity, socioecological equilibrium, stewardship, and transdisciplinarity. The application and effectiveness of the definition are incomplete without the monitoring and evaluation of these basic principles. The presented definition also reinforces the overall aims of related concepts, particularly Eco-Health (by highlighting the ecocentric versus anthropocentric scope) and Planetary Health (explicitly acknowledging the relevance of environmental/ecosystem health).

**Box 1. One Health definition and key underlying principles**

**Definition**

**One Health** is an integrated, unifying approach that aims to sustainably balance and optimize the health of people, animals, and ecosystems. It recognizes the health of humans, domestic and wild animals, plants, and the wider environment (including ecosystems) are closely linked and interdependent.

The approach mobilizes multiple sectors, disciplines, and communities at varying levels of society to work together to foster well-being and tackle threats to health and ecosystems, while addressing the collective need for healthy food, water, energy, and air, taking action on climate change and contributing to sustainable development.

**Key underlying principles including**

1. **equity** between sectors and disciplines;
2. sociopolitical and multicultural **parity** (the doctrine that all people are equal and deserve equal rights and opportunities) and inclusion and engagement of communities and marginalized voices;
3. socioecological **equilibrium** that seeks a harmonious balance between human–animal–environment interaction and acknowledging the importance of biodiversity, access to sufficient natural space and resources, and the intrinsic value of all living things within the ecosystem;
4. **stewardship** and the responsibility of humans to change behavior and adopt sustainable solutions that recognize the importance of animal welfare and the integrity of the whole ecosystem, thus securing the well-being of current and future generations; and
5. **transdisciplinarity** and multisectoral collaboration, which includes all relevant disciplines, both modern and traditional forms of knowledge and a broad representative array of perspectives.

The newly formed operational OHHLEP definition aims to be comprehensive, to promote a clear understanding across sectors and areas of expertise, and to support the Partners and their Member States in framing their One Health strategies, programs, and implementation plans. This includes the forthcoming Joint Plan of Action for One Health (2022 to 2026), the key strategic framework that will guide the cross-sectoral collaborative activities of FAO, OIE, UNEP, and WHO. The definition should be considered as an overarching set of guiding principles that can be further tailored to specific stakeholders. It is intended to assist in orienting the general outline and considerations for a One Health approach and the opportunities for innovation, cooperation, and collaboration among all relevant sectors and disciplines. While food and water security, energy, and environmental/ecosystem health are wider topics with sector-specific and specialist concerns that may extend beyond the scope of One Health approaches, their interface is where multiple sectors have shared responsibility and relevance in protecting health and addressing health challenges. This One Health approach is not just focused on zoonotic disease or antimicrobial resistance but can address the full spectrum from prevention, health improvement, and health promotion to the detection, preparedness, response, and recovery from health crises. The approach is applicable at community, subnational, national, regional, and global levels. It relies on shared and effective governance, communication, collaboration, and coordination to understand co-benefits, risks, trade-offs, and opportunities for equitable and holistic solutions.

Political commitment and leadership, including prioritization and allocation of resources that are distributed in equitable ways, are essential for the successful implementation of the integrated One Health vision. However, we need to recognize that there are substantial political, legal, ethical, financial, capacity, and societal barriers and complexities in developing and implementing a unified One Health approach. The commitment of political, sectoral, organizational, and individual societies to implement it successfully will address human, animal, and ecosystem health, including other issues like biodiversity loss, clean air and energy, the impact of climate change, food and water security, and social inequalities. This approach has clear advantages to improve health for all, embed social and environmental protection, and support sustainable economic development and resilience.

